# Virome Analysis of Lemon Plants with Vein Clearing Symptoms Reveals Mixed Infection of Citrus Vein Clearing Virus, Iris Domestica Betaflexivirus 1 and Hop Stunt Viroid

**DOI:** 10.3390/v18010141

**Published:** 2026-01-22

**Authors:** Myeonghwan Kwak, Eui-Joon Kil, Angelo De Stradis, Giuseppe Parrella

**Affiliations:** 1 Department of Plant Medicals, Andong National University, Andong 36729, Republic of Korea; kwak2604@gmail.com (M.K.); viruskil@gknu.ac.kr (E.-J.K.); 2 Institute for Sustainable Plant Protection of the National Research Council (IPSP-CNR), 70126 Bari, Italy; 3 Institute for Sustainable Plant Protection of the National Research Council (IPSP-CNR), 80055 Portici, Italy

**Keywords:** CYCVC, IDBV, HSVd, *Citrus lemon*, high-throughput sequencing, emerging viruses

## Abstract

Citrus yellow vein clearing virus (CYVCV) is the causative agent of the yellow vein clearing disease (YVCD), a worldwide and highly destructive disease in lemon (*Citrus lemon*) and sour orange trees (*C. aurantium*). The typical symptoms of vein clearing are believed to be associated with CYVCV infection in citrus, so virus-specific diagnostic systems are currently used to confirm infection. In the present study, virome analysis based on high-throughput sequencing (HTS) on a lemon plant showing YVCD revealed mixed infection of CYVCV, iris domestica betaflexyviridae 1 (IDBV), and hop stunt viroid (HSVd). This multiple infection was confirmed in other two lemon plants with similar symptoms using virus/viroid specific primers. This is the first report of IDBV in lemon. Through molecular characterization and the reconstruction of phylogenetic relationships, a possible origin of the viruses/viroid identified in lemon has been hypothesized. Such mixed infections raise new questions about their role in the expression of YVCD symptoms observed on lemon.

## 1. Introduction

Citrus yellow vein disease (CYVD) is an emerging disease of citrus that was first observed in Pakistan in 1988 in lemon (*Citrus lemon*) and sour orange trees (*C. aurantium*) [1]. Citrus yellow vein clearing virus (CYVCV), a member of the virus subgenus *Mandarivirus*, genus *Potexvirus*, family *Alphaflexiviridae*, was identified as the causal agent of CYVD [2] and until today it has been detected in Pakistan, India, China, Iran, Türkiye, Korea, USA, and Italy [3]. The rapid spread of CYVCV in China since 2009 has significantly affected lemon production, with citrus trees like lemon and sour orange experiencing stunted growth, reduced yields, yellow vein clearing, leaf deformities, intermittent ringspots, and venial necrosis [4]. Consequently, CYVCV poses a significant threat to the citrus industry worldwide, and since October 2022, it has been included in the European Plant Pathology Organization (EPPO) alert list (November 2025), which reports all pests that may present a phytosanitary risk for the EPPO region. Nevertheless, recently it has been demonstrated that a different virus-like agent, named citrus yellow vein associated virus (CYVaV), identified by high-throughput sequencing (HTS) in an old citrus sample date back 1969, was also capable of inducing yellow vein symptoms independently of other known citrus viruses or viroids [5].

Over the past decade, virologists have discovered an unprecedented number of viruses using HTS, leading to advancements in understanding the diversity of viruses in nature and the virome of agricultural crops. Moreover, these discoveries often widen gaps in our understanding of virus biology, particularly the role of new viruses in disease. Moreover, HTS is a powerful tool for obtaining information about plant virome associated to particular symptoms. Thus, when used critically in etiological studies, HTS is a powerful means to establish disease causality between the virus and its host [6].

In spring 2024, during a visual inspection in a nursery located in the Lazio region (central Italy), some lemon plants were identified with suspicious symptoms of CYVD, consisting of vein clearing and deformations of the leaves resembling those caused by CYVCV. However, through HTS analysis these plants resulted unexpectedly mixed infected by CYVCV, iris domestica betaflexivirus 1 (IDBV), and hop stunt viroid (HSVd).

## 2. Materials and Methods

### 2.1. Plant Material and Electron Microscope Observations

Apical leaves showing CYVD symptoms were collected from 4–5-year-old potted lemon plants (*n* = 3) identified in 2024 in a commercial nursery located in province of Latina (Lazio Region, central Italy). Plant sap from these symptomatic leaves were used for dip assays (dip method) and for subsequent molecular analyses.

Briefly, a small piece of symptomatic tissue was placed on a glass slide and a few drops of sample buffer, composed of 0.1 M Na_2_H/KH_2_PO_4_ buffer (pH 7.0), 2% (*w*/*v*) polyvinyl pyrrolidone (PVP, MW 11.000), and 0.2% (*w*/*v*) Na_2_SO_3_, were added. The leaves were cut into small pieces with a razor blade in the buffer and the plant sap was used to make adsorbed preparations. Carbon-coated grids of 400 mesh were incubated for 5 min with a drop of the homogenates prepared from virus-infected plant samples and subsequently washed with a gentle stream of approximately 20 drops of deionized water (Milli Q) to remove buffer salts. For negative staining, 3–5 drops of a 1% uranyl acetate solution in sterile deionized water were applied. The grids were then dried by gentle tapping on a piece of Whatman paper and examined with transmission electron microscope [7,8].

### 2.2. Preliminary Molecular Screening for CYVCV

**Figure 1:**
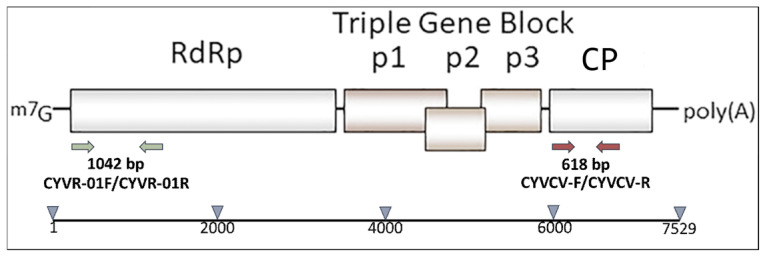
Schematic diagram of genomic structure of CYVCV indicating positions of primer pair CYVR-01F/CYVR-01R (green arrows), within RdRp ORF, and CYVCV-F/CYVCV-R (red arrows), within CP ORF, used in this study for CYVCV preliminary screening.

Total RNAs were extracted with the E.Z.N.A.^®^ Plant RNA Kit (Omega Bio-tek, Norcross, GA, USA), and subjected to RT-PCR using the CYVCV specific primer pair CYVR-01F/CYVR-01R [9], designed to amplify a portion of about 1000 bp at the 5′ end of the RdRp gene (Figure 1, green arrows), and the new primer pair CYVCV-F (5′-TACCGCAGCTATCCATTTCC-3′) CYVCV-R (5′-GCAGAAATCCCGAACCACTA-3′) designed to amplify a portion of ca. 600 bp of the coat protein (CP) gene (Figure 1, red arrows). A volume of 2 μL of RNA was reverse transcribed at 42 °C in a final volume of 20 μL.

PCR amplification was performed in volumes of 25 μL, each containing 2.5 μL of the template genomic DNA, 12.5 pmol of each primer, 12.5 μL of GoTaqGreen Master Mix (Promega, Madison, WI, USA), and 9.5 μL of sterile distilled water, using a Mastercycler Nexus X2 (Eppendorf, Hamburg, Germany) thermocycler, under the following conditions: 3 min at 94 °C as pre-denaturation, thermal cycling for 35 cycles (50 s at 94 °C, 50 s at 55 °C, and 1 min at 72 °C), and 10 min at 72 °C as final extension. PCR products were visualized on 1.2% agarose gel and stained with ethidium bromide. Amplicons of the expected size obtained from symptomatic leaf samples were sequenced at Microsynth Seqlab (Göttingen, Germany) to confirm the identity of the virus. Blast analysis of the sequences obtained was performed online (http://blast.ncbi.nlm.nih.gov/Blast.cgi, accessed on 16 September 2025).

### 2.3. High-Throughput Sequencing and Bioinformatic Analysis

For HTS analysis total RNA extraction was carried out with TRI-reagent (Sigma-Aldrich, St. Louis, MO, USA) using 100 mg of fresh leaves. Ribosomal RNA was depleted from the purified RNA using the RiboMinus Plant Kit for RNA-Seq (Thermo Fisher Scientific, Waltham, MA, USA), and sequencing libraries were prepared by using TruSeq stranded total RNA with the RiboZero Plant kit (Illumina, San Diego, CA, USA). High-throughput sequencing was performed on the Illumina NovaSeq 6000 platform, with 150 nt paired-end chemistry by Macrogen company (Seoul, Republic of Korea). The bioinformatics analysis of the obtained raw data was performed using the CLC Genomics Workbench (Qiagen, Hilden, Germany). The reads obtained were trimmed (reads with *Q* ≤ 25 and shorter than 15 bp were discarded) and host transcripts were filtrated using collection reads that were not mapped to the host genome (CGD21001). Filtered sequencing reads were assembled into contigs using *de novo* assembly, with a minimum contig length cutoff of 200 bp. The trimmed sequencing reads were first mapped to NCBI viral RefSeq database (September 2024). Additionally, *de novo* assembly was further performed to detect possible novel and/or variant viruses, and the assembled contigs were annotated by BLASTn search to NCBI GenBank. The reads were then mapped to the corresponding most similar viral genome sequences to the contigs from NCBI GenBank. Scaffolds were finally assembled by mapping the contigs and residual reads on viral reference genomes. Finally, the consensus viral genomes were deposited in NCBI GenBank and used for further analyses.

### 2.4. Validation of HTS Data by RT-PCR

Additional specific primers were also designed to confirm the presence of the viruses and viroid detected by HTS in the original lemon plant and in the other two different lemon plants showing YVCD, identified in the same nursery. The cDNAs were produced as described above (see Section 2.2), while PCR amplifications were performed using the proofreading Platinum SuperFi II DNA Polymerase (Thermo Fisher Scientific Inc., Waltham, MA, USA) under the following cycling conditions: initial denaturation at 94 °C for 4 min; 35 cycles of 94 °C for 30 s, 56 °C for 30 s, and 72 °C for 1 min; and final extension step at 72 °C for 10 min. All PCR products were visualized on 1.2% agar gels stained with ethidium bromide and then directly sequenced in both senses (Microsynth, Seqlab GmbH, Göttingen, Germany).

### 2.5. Construction of Phylogenetic Neighbor Network, Maximum-Likelihood Tree and Further Analysis

The complete genomes different isolates of CYVCV, HSVd, and IDBV were acquired from GenBank sequence database. Sequences of each virus/viroid were aligned, including the sequences assembled in this study, using MEGA 11 with the MUSCLE algorithm [10]. In total, 80 CYVCV, 67 HSVd, and 17 IDBV sequences were compared. The construction of a neighbor network was executed and subsequently modified utilizing SplitsTree 4, with 1000 bootstrap replicates [11]. Phylogenetic trees were inferred using the maximum-Likelihood (ML) method implemented in the MEGA software (version 11.0) [10]. Evolutionary distances were calculated using the Kimura two-parameter model [12]. Bootstrap values based on 1000 replications were incorporated to assess the statistical support for the tree topologies [13].

The recombination analysis of complete genomes of 80 CYVCV, 17 IDBV, and 67 HSVd isolates was conducted using the Recombination Detection Program v4.56 (RDP4) software [14], using various algorithms, including RDP, GENECONV, CHIMAERA, MAXCHI, BOOTSCAN, SISCAN, and 3SEQ. Only recombination events detected by at least four different methods were considered as potentially true recombination breakpoints.

HSVd secondary structure was predicted with the aid of the *mfold* software (https://www.unafold.org/mfold/applications/rna-folding-form.php, 16 September 2025, circular version) and compared with those described previously [15].

## 3. Results

### 3.1. Symptoms and Electron Microscopy

**Figure 2:**
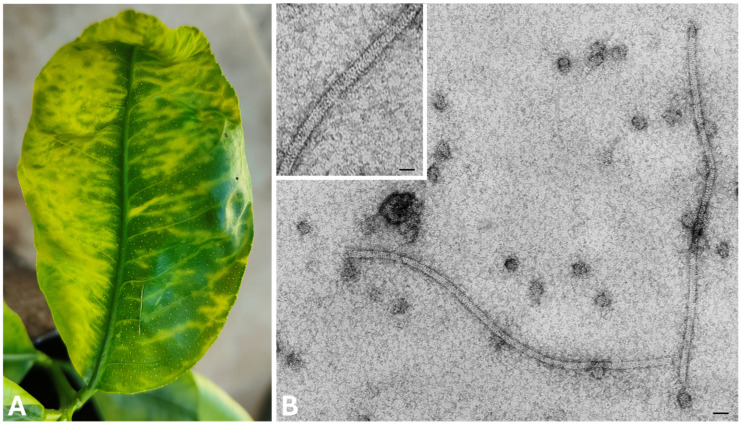
(**A**) Symptoms observed on youngest apical leaves of infected lemons; (**B**) flexuous and filamentous virus particles of about 685 nm in length observed in leaf dip preparations from the same leaves (bar represents 50 nm in main photo and 25 nm in insert).

Symptoms observed on the leaves of lemon plants consisting of vein clearing and deformations of the leaves (Figure 2A), resembling those caused by CYVCV (genus *Mandarivirus*, family *Alfaflexiviridae*) in citrus species. Symptoms were mostly concentrated in the leaves of the distal portion of the branches. The electron microscopy examination of leaf dip extracts obtained from these leaves, showed constantly the presence of filamentous virions of about 685 nm in length and 14–15 nm in diameter (Figure 2B), resembling those described for the “*flexiviridae*” families within the order *Tymovirales* [2,16].

### 3.2. Detection of CYVCV by RT-PCR

**Figure 3:**
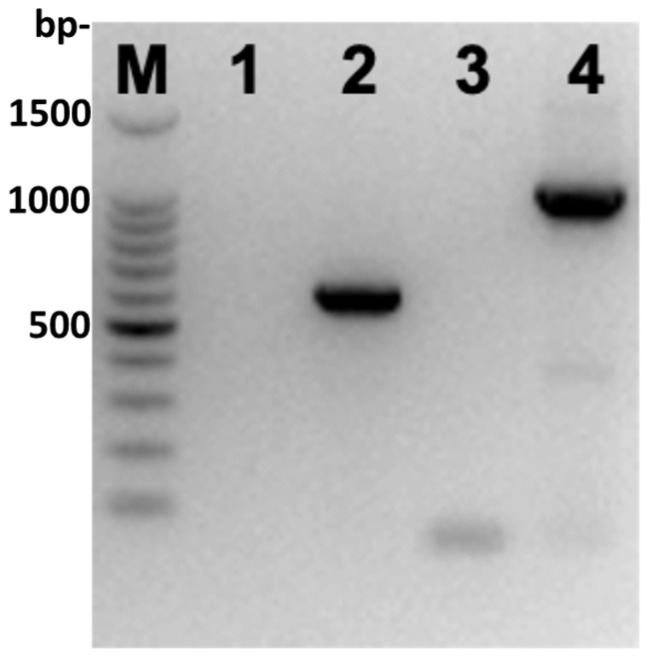
Agarose gel electrophoresis of the RT-PCR products obtained with the primers CYVCV-F/CYVCV-R (this study; lane 2) and CYVR-01F/CYVR-01R (lane 4) [9], using RNA extracted from symptomatic leaves. M = 100 bp DNA ladder (Promega, USA); Lanes 1 and 3 = negative controls (healthy plant).

Amplicons of the expected size obtained from symptomatic leaf samples (Figure 3) were sequenced at Microsynth Seqlab (Göttingen, Germany) to confirm the identity of the virus. The sequence of the amplicon obtained with the primers CYVR-01F/CYVR-01R showed highest percentage of nucleotide identity (97.6%) with the Indian isolate CYVCV-Kin-Del-Dec-2022 (Acc. No. OR251443) and Chinese isolate ZJ_2 (Acc. No. KY933795). The sequence of the amplicon obtained with the primers CYVCV-F/CYVCV-R showed the highest percentage of nucleotide identity (97.9%) with the isolates CA4 (Acc. No. OQ418492) and C16 (OQ418501) both from California (USA).

### 3.3. HTS Results and Validation of Identified Viruses

The RNA extracted from the leaves of a symptomatic lemon plant passed the quality control (QC) showing an RNA integrity number (RIN) of 5.3. To guarantee reliability of the data, QC was assessed at each step of the procedure. A total of 62,497,250 reads of sequence data was obtained from Illumina sequencing of constructed cDNA libraries, with 62,497,250 raw reads and 6,312,222,250 bases. After trimming, 62,495,454 trimmed reads and 6,310,165,990 bases were obtained, with quality scores of Q30 and 49% GC content. Of these, 217,020 reads were related to viruses (Supplementary Figure S1A). Overall, the raw data with average length of 101 bp became of 100.97 bp after trimming. *The de novo* assembly and BLASTn results generated three contigs related to partial/near-complete genomes of these viruses/viroid: one related to CYVCV, one related to IDBV, and one related to HSVd, respectively (Supplementary Figure S1A). The presence of viruses identified by HTS in the lemon sample were validated by RT-PCR in the same sample, using specific primers for each viruses/viroid, and amplified amplicons were Sanger sequenced to confirm the identity of the viruses (Supplementary Figure S1B). In addition, other two lemon plants showing YCVD, identified the same nursery, gave positive results by RT-PCR using same specific primers. Amplicons of the expected size, specific for each virus/viroid, were sequenced, and sequences were 100% identical to the corresponding regions obtained by HTS sequencing, confirming presence of CYVCV, IDBV, and HSVd in the three lemon plants.

### 3.4. Molecular Characterization and Phylogenetic Relationships

The genome of the CYVCV isolate was found to be 7530 bp in length, with an average G+C content of 51.76%. The genome sequence data has been deposited in GenBank under the accession number PV870367. The first 79 nt, corresponding to the 5′-untranslated region (UTR), showed the highest percentage of identity (98.73%) with isolates (eight out of nine) belonging to the South Asia, Middle East, North America group. The 3′ UTR was of 37 nt, excluding the 3′poly-A tail, and showed 100% of identity with almost all isolates present in GenBank. Sequence analysis suggested that the CYVCV Italian isolate has six open reading frames (ORFs; Supplementary Figure S2) encoding six different putative proteins with molecular weight of 187, 25, 12, 6.4, 34, and 23 kD, which were thought to have different functional activities [2]. The genome shared 97.5% of nucleotide identity with a previously described CYVCV isolate (MF563877; Supplementary Figure S2). Overall, molecular features of the six ORFs of the Italian CYVCV isolate are similar with those described for the reference Turkish isolate CYVCV-Y1 belonging to the South Asia, Middle East, North America group (Acc. N. JX040635) [2].

**Figure 4:**
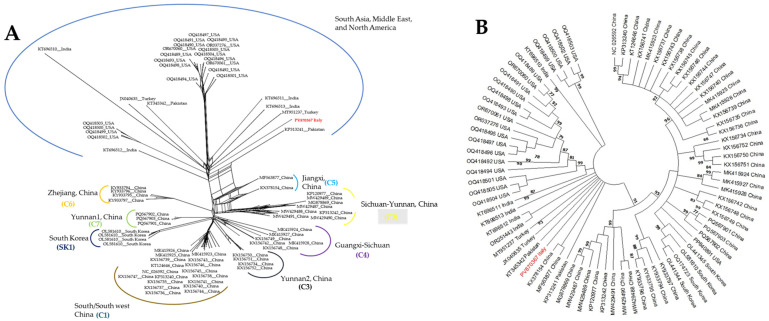
Phylogenetic reconstruction of non-rooted trees based on CYVCV sequences. (**A**) Neighbor network reconstruction of complete genomes of 80 CYVCV isolates. The genetic groups are highlighted with lines of different colors; (**B**) Maximum-likelihood consensus tree obtained using complete genome of 80 CYVCV isolates, adopting Kimura 2-parameters and 1000 bootstraps replication. Position of Italian isolate is marked red in both trees.

Phylogenetic analysis using a neighbor-net reconstruction of CYVCV complete genomes unveiled two major genotype groups as previously reported [17] (Figure 4A), with the CYVCV isolates from China and South Korea formed a major group termed the “East Asia group”, composed by eight subgroups, one from Korea (SK1) and seven from China (C1–C7); while isolates from India, Pakistan, Türkiye, and California/USA formed a second major genotype, namely the “South Asia, Middle East, North America group”. The isolate from Italy shown to be more related to isolates from Türkiye and Pakistan within the “South Asia, Middle East, North America group” (Figure 4A). Maximum-likelihood consensus tree placed the CYVCV Italian isolate more ambiguously between Chinese and Pakistani isolates (Figure 4B).

A complete or near-complete genome of 4985 nt of a IDBV isolate was reconstructed after HTS analysis (Supplementary Figures S1A and S2) and the presence of this virus was validated by RT-PCR (Supplementary Figure S1B). The genome sequence data has been deposited in GenBank under the accession number PV870368. The genome shared 95% of nucleotide identity with a previously described IDBV isolate (PP274697; Supplementary Figure S2) and was composed of a single ORF containing viral methyltransferase, amino acid positions (aap) 48–342 (pfam01660, E-value = 5.96 × 10^−50^), viral helicase1, aap 790–1038 (pfam01443, E-value= 6.93 × 10^−20^), and Betaflexiviridae RNA-dependent RNA polymerase, aap 1236–1553 (cd23245, E-value= 1.86 × 10^−179^) motifs (Supplementary Figure S3).

**Figure 5:**
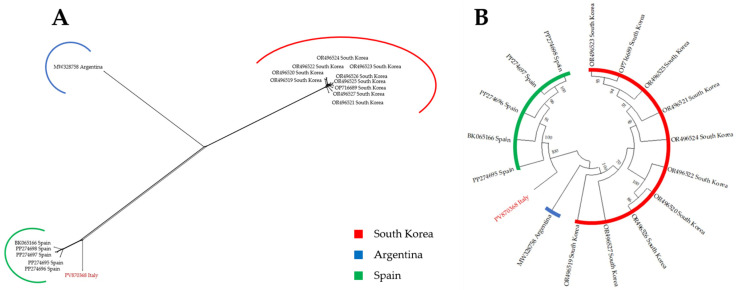
Phylogenetic reconstruction of non-rooted trees based on iris domestica betaflexiviridae (IDBV) sequences. (**A**) Neighbor network reconstruction of complete genomes of 17 IDBV isolates. (**B**) Maximum-likelihood consensus tree obtained using complete genome of 17 IDBV isolates, adopting Kimura 2-parameters and 1000 bootstraps replication. Position of Italian isolate is marked red in both trees.

Phylogenetic analysis using a neighbor-net reconstruction of IDBV complete genomes showed that the IDBV isolate detected in lemon, although distinct from the other isolates described so far, was more related to a group of isolates from Spain (Figure 5A), allowing a clearer placement compared to the phylogeny obtained with ML (Figure 5B).

In addition to CYVCV and IDBV, HTS identified also an isolate of HSVd with a genome length of 300 nt that has been deposited in GenBank under the accession number PV870369. The genome shared 99% of nucleotide identity with a previously described HSVd isolate (MT155390; Supplementary Figure S2). Based on the primary and predicted secondary structure, this isolate was classified as ‘non-cachexia’ variant of HSVd since the specific nucleotide differences that determine expression of cachexia symptoms (“cachexia expression motif”) were not present in the ‘variable domain’ [15] (Figure S4).

**Figure 6:**
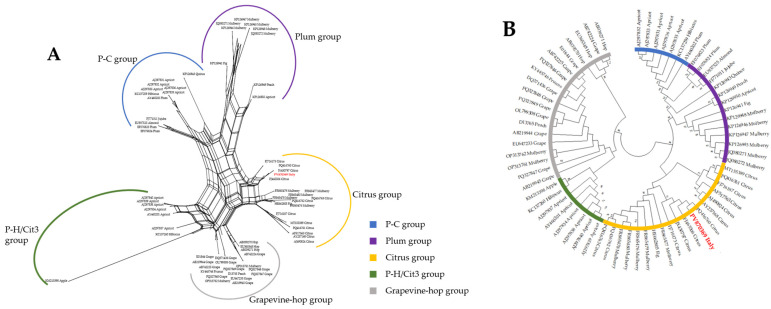
Phylogenetic reconstruction of non-rooted trees based on hop stunt viroid (HSVd) sequences. (**A**) Neighbor network reconstruction of complete genomes of 67 HSVd isolates. (**B**) Maximum-likelihood consensus tree obtained using complete genome of 67 HSVd isolates, adopting Kimura 2-parameters and 1000 bootstraps replication. Position of Italian isolate is marked red in both trees.

From phylogenetic point of view, both neighbor network and ML reconstruction clearly placed the new HSVd isolate within non-recombinant ‘Citrus group’ (Figure 6).

## 4. Discussion

CYVCV is a positive-sense single-stranded virus that belongs to genus *Mandarivirus* in the family *Alphafexiviridae* [2]. CYVCV virions are flexible filamentous particles, with a diameter of 13 to 14 nm and a modal length of 685 nm CYVCV is an important viral disease affecting citrus plants, causing significant reduction of production in citrus-producing areas globally. Lemon yield losses have reached 50–80% in China, highlighting the economic effects of the virus. CYVCV isolates have been found in seven countries, including India, Pakistan, China, South Korea, Iran, Türkiye, and California, USA. Although it was first noted in Pakistan in 1988, complete genome sequencing was not done until 1992.

Although CYVCV was previously reported in Italy [18], this new report presents the first complete genome sequence of a CYVCV isolate from Italy, obtained from a lemon plant showing typical CYVCV leaf symptoms (Figure 1), using HTS technology. Neighbor network analysis of full genome sequences of CYVCV isolates revealed closer genetic ties between the Italian isolate with those from India, Pakistan, and Türkiye compared to those from China and South Korea, suggesting a possible origin of the Italian isolate (Figure 4A).

Unexpectedly, in addition to CYVCV, HTS analysis revealed co-infection with IDBV and HSVd in the same plant, and such triple mixed infection was confirmed by RT-PCR in two other different lemon plants showing similar symptoms. Although the detection of HSVd is not surprising, since HSVd is one of the main viroids circulating in all citrus-growing areas worldwide [19], IDBV is a novelty in lemon since it has been described so far in *Iris domestica* in Argentina (direct submission: MW328758.1), *Cnidium officinale* in South Korea (direct submission: OP716689; OR496519-27), and *Crocus sativus* [20]. Based on the IDBV genomic sequences available in GenBank, phylogenetic network showed that this new isolate is closely related, although distinct, to Spanish isolates from saffron (*Crocus sativa*), raising the question about the origin of such isolate (Figure 5A).

Interestingly, and at the same time difficult to explain, the genome of this new Italian IDBV isolate contains only the ORF of the RNA-dependent RNA polymerase (RdRp) (Figures S2 and S3). Nonetheless, our results are perfectly in agreement with those regarding the identification and characterization of all IDBV isolates described so far, whose sequences have been the subject of studies published in scientific journals [20] or directly deposited in GenBank. Furthermore, IDBV belongs to the group of “unclassified *Betaflexiviridae*”, among which are found other viruses with the same characteristic as IDBV (i.e., the genome contains only the ORF of the RdRp), which have also been the subject of extensive publication [21]. This scenario could be the result of complex interactions between viruses such as functional complementation and synergism. Functional complementation in plant viruses involves one virus supplying a missing protein (like a movement or silencing suppressor protein) that enables another virus, often defective or from a different genus, to overcome a host restriction, facilitating its replication, cell-to-cell movement, or systemic spread, leading to synergistic infections or novel functionalities [22]. This often occurs in co-infections mainly with related viruses (but not necessarily), where a “helper” virus provides a missing function, such as movement protein (MP) or silencing suppressor, allowing a dependent virus to move through a host that would otherwise block it, expanding the host range of the dependent virus or creating severe symptoms [22]. This could explain why the “unclassified *Betaflexiviridae*” with “incomplete” genome, were always found in mixed infections with other “complete” viruses belonging to the Alphaflexiviridae and/or Betaflexiviridae families. For example, IDBV was found associated with safron betaflexivirus 1 in *Crocus sativus* and other alphaflexiviruses [20], while sisal-associated betaflexivirus A, sisal-associated betaflexivirus B, sisal-associated betaflexivirus C, and sisal-associated betaflexivirus E, all of them with incomplete genome as IDBV, were found associated with cowpea mild mottle virus (CMMV, genus *Crinivirus*, family *Betaflexiviridae*) in sisal (*Agave* spp.) plants [21].

Based on current data, it is not known how widespread IDBV is in the lemon germplasm or our data represent an exceptional finding. It is well known that high-throughput sequencing (HTS) offers several advantages for virus detection, including the ability to find novel and unexpected viruses without prior knowledge, high sensitivity for detecting low-prevalence variants, and comprehensive, high-throughput analysis from a single sample. This allows for simultaneous detection of multiple viruses and rapid characterization of viral genomes for tracking evolution as well. Moreover, it outperforms traditional methods by providing a more complete picture of viral infections, reducing the time and cost per sample, and allowing for ongoing re-analysis as databases expand.

Although disease symptoms and intensities of CYVCV vary significantly depending on virus strains, citrus varieties, and environmental conditions [23,24], typical vein clearing symptoms are considered associated to CYVCV infection, orientating the diagnostics to the direct use of virus-specific diagnostic methods. After the determination of the first complete genomic sequence of a CYVCV isolate from Türkiye [2], due to the typical foliar symptoms the presence of the virus in symptomatic plants has almost always been confirmed by RT-PCR using specific primers and subsequent Sanger sequencing of the obtained amplicons or by specific RT-qPCR. As is well known, such an approach, although it ensures rapid confirmation of the presence of CYVCV in plants with typical symptoms, does not allow for the verification of other possible associations, such as the presence of other viruses/viroids, as reported in this study.

It is unknown whether CYVCV and IDBV are spreading in lemon germplasm, neither if this mixed infection could reflect CYVCV symptoms variability reported [23,24]. Thus, a broader and more in-depth studies are needed. Using HTS to determine the virome of lemon would allow us to know the extent of their distribution, the diversity of possible new variants of these viruses in this plant, the damage they cause, and perhaps their possible relationship with other species of citrus or even wild plants. As result, knowledge about the viruses that infect this important crop will benefit future assessments of its phytosanitary status and production and certification strategies.

Our study provides new insights for advancing our comprehension of lemon viruses and extending the spectrum of viruses that could potentially pose a threat to this cultivation. Nevertheless, definitive studies are needed to understand the effects of interactions in such mixed infections and in particular the possible role in the observed symptoms.

## Data Availability

Data are contained within the article and Supplementary Materials.

## Supplementary Materials

The following supporting information can be downloaded at: https://www.mdpi.com/article/10.3390/v18010141/s1, Figure S1: Workflow of the entire virome analysis of a symptomatic *Citrus lemon* plant; Figure S2: Genome organization of the viruses detected and average coverage, length in base pair and accession numbers with the highest percentage of nucleotide identity with the assembled virus sequences; Figure S3: Identification of conserved domains in the protein of the IDBV isolate identified in lemon by CD-search; Figure S4: Comparison of the primary and secondary structures of the HSVd cachexia expression motif between HSVd isolate identified in the present study (PV870369) and cachexia (AF213484) and non-cachexia (AF213503) reference strains.
