# Supplementary material for: Virome Analysis of Lemon Plants with Vein Clearing Symptoms Reveals Mixed Infection of Citrus Vein Clearing Virus, Iris Domestica Betaflexivirus 1 and Hop Stunt Viroid
Source: Viruses. 2026 Jan 22;18(1):141. doi: 10.3390/v18010141 (PMC12846569; doi:10.3390/v18010141)

Supplementary materials

Figure S1 - Workflow of the entire virome analysis of a symptomatic *Citrus lemon* plant (A) and RT-PCR validation of the viruses detected by HTS (B). + = symptomatic lemon sample; - = healthy lemon sample; M = 100 bp DNA ladder (Bioneer, Republic of Korea)

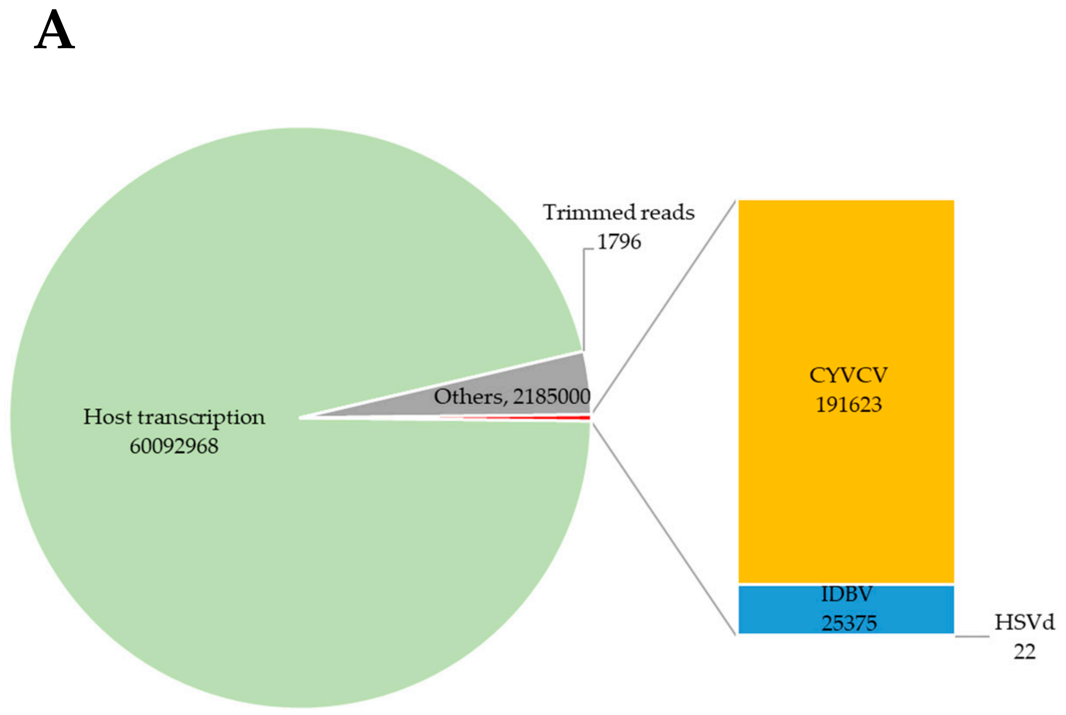

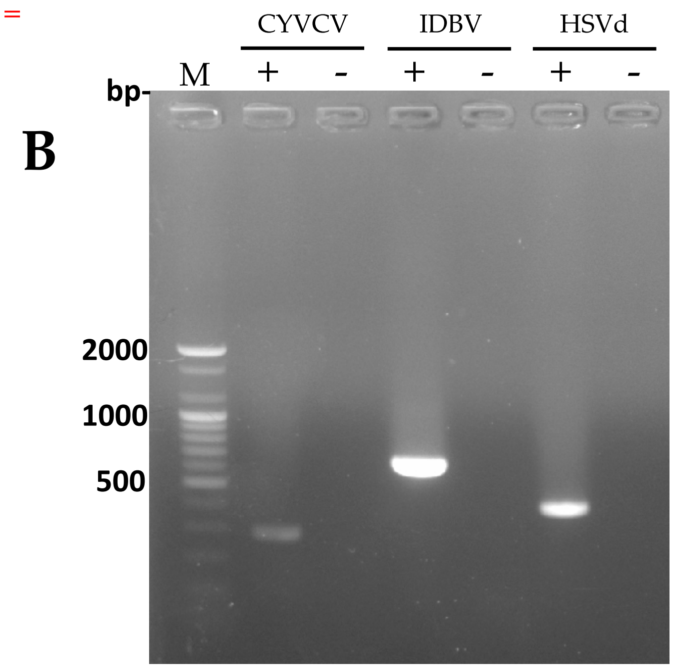

Primer name	5'- sequence -3'	Size
HSVd_f(1155)	GTTCGTCTTCCAACCTGCTTT	274
HSVd_r(1155)	TCACTCGAAGAGCCAGGAGAA	
CYVCV_f(1155)	GCTTTCCACGCTGAAGCAAA	632
CYVCV_r(1155)	CCTTTGGTGAAGGCAACGTG	
IDBV1_f(1155)	TGGGAATAACGAAGGTGCCC	407
IDBV2_r(1155)	TTCGCAGATGTTGTCCACGA	

Figure S2 – Genome organization of the viruses detected and average coverage, length in base pair and accession numbers with the highest percentage of nucleotide identity with the assembled virus sequences. For citrus yellow vein clearing virus ORFs are: RdRp=RNA-dependent RNA polymerase, TGB=Triple Gene Block, NaBP=nuceic acid-binding protein; for Iris domestica betaflexivirus 1 only one ORF have been identified, corresponding to the RdRp; for hop stunt virois structural domains are: TL=Terminal Left, P=Pathogenic, C=Central; V=variable, TR=Terminal Right.

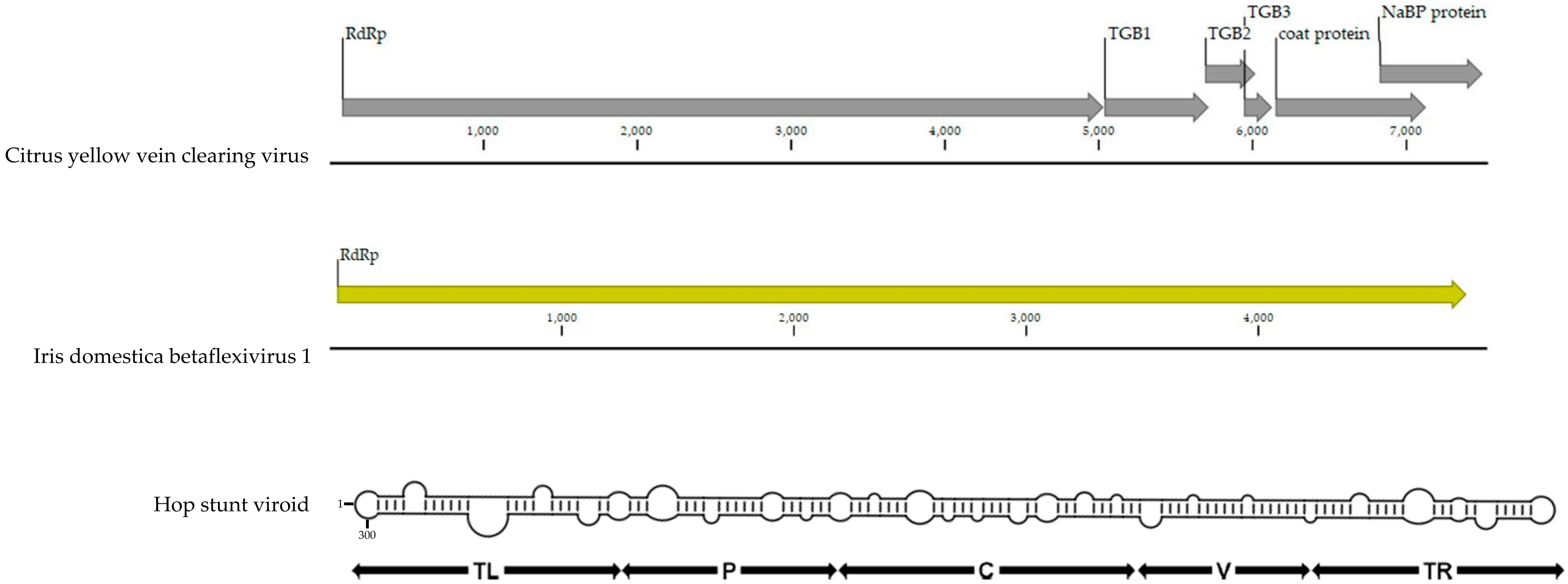

Family	Genus	speices	Average coverage	Length (bp)	Identity %	(acession number)
Alphaflexiviridae	Potexvirus	Citrus yellow vein clearing virus	2,555.76	7530	97.48	(MF563877)
Betaflexiviridae	Betaflexivirus	Iris domestica betaflexivirus 1	509.21	4985	95.05	(PP274697)
Pospiviroidae	Hostuviroid	Hop stunt viroid	7.1	300	99.01	(MT155390)

Figure S3. Identification of conserved domains in the protein of the IDBV isolate identified in lemon by CD-search (https://www.ncbi.nlm.nih.gov/Structure/cdd/wrpsb.cgi)

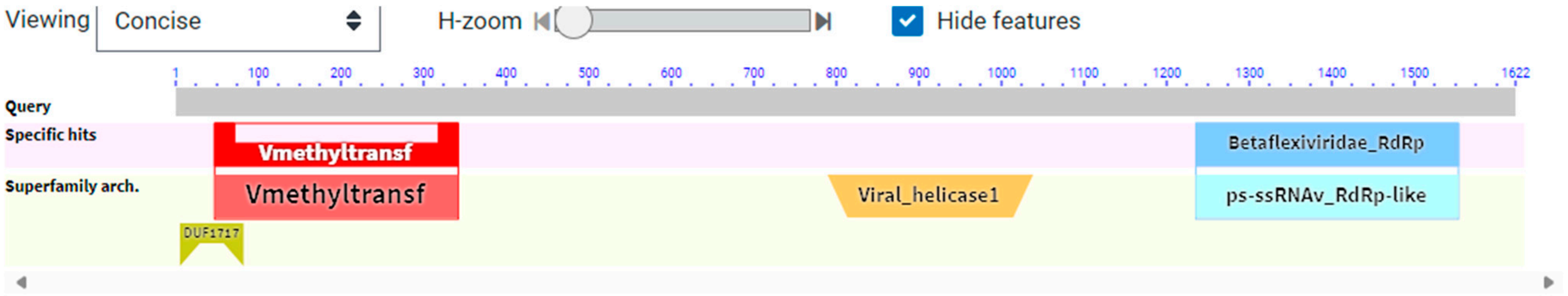

Name	Accession	Description	Interval	E-value
+ Betaflexiviridae_RdRp	cd23245	catalytic core domain of RNA-dependent RNA polymerase (RdRp) in the family Betaflexiviridae of positive-sense single-stranded RNA [(+)ssRNA] viruses, in the order Tymovirales	1236-1553	1.86×10^{-179}
+ Vmethyltransf	pfam01660	Viral methyltransferase	48-342	5.96×10^{-50}
+ Viral_helicase1	pfam01443	Viral (Superfamily 1) RNA helicase	790-1038	6.93×10^{-20}
+ DUF1717	pfam05414	Viral domain of unknown function (DUF1717)	6-82	3.00×10^{-3}

Figure S4. Comparison of the primary and secondary structures of the HSVd cachexia expression motif between HSVd isolate identified in the present study (PV870369) and cachexia (AF213484) and non-cachexia (AF213503) reference strains.

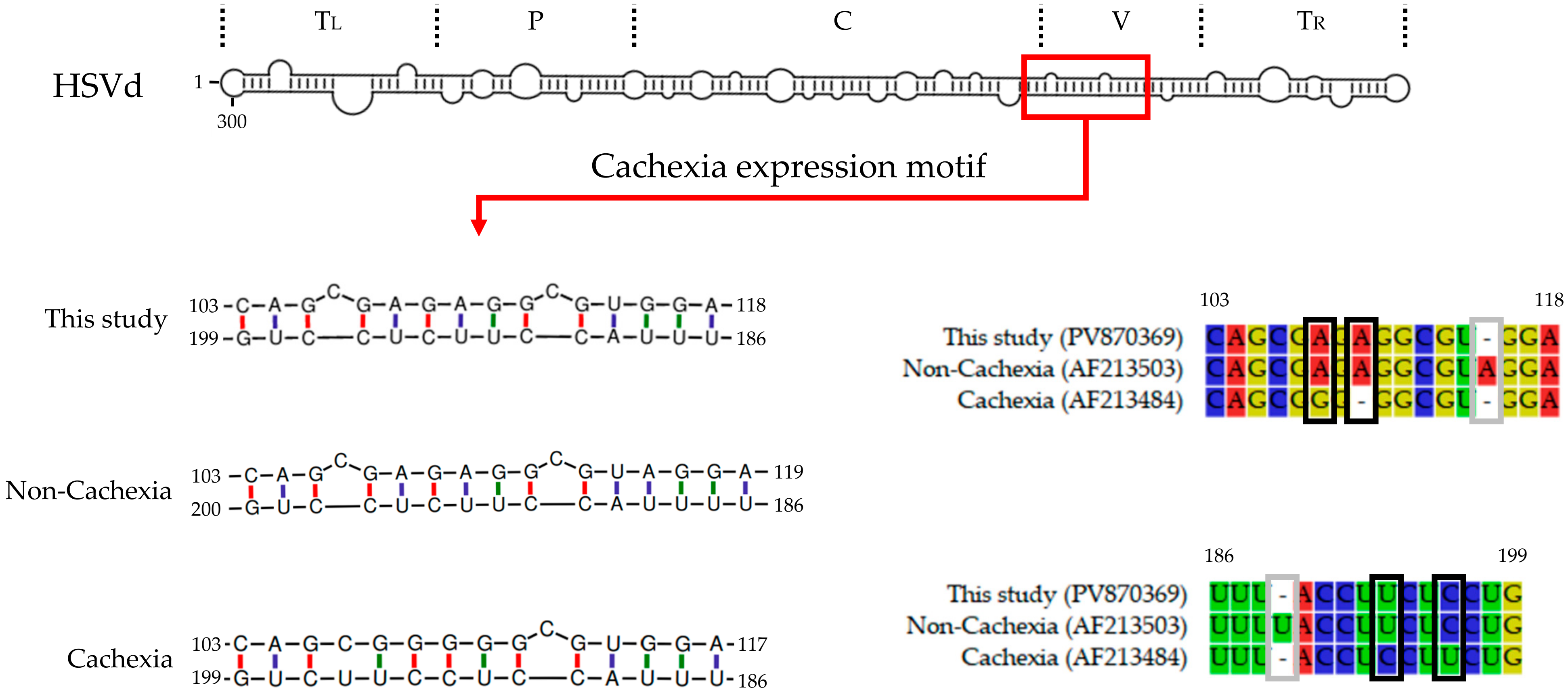

Supplement: Supplementary file 1 [file viruses-18-00141-s001.zip › viruses-4080930-supplementary.pdf]
